# Acquired Methemoglobinemia in a Ketamine-induced Ulcerative Cystitis Patient: A Case Report

**DOI:** 10.5811/cpcem.2022.1.55277

**Published:** 2022-03-07

**Authors:** Spencer Kozik, Cali Kirkham, Gabriel Sudario

**Affiliations:** * University of California – Irvine, School of Medicine, Irvine, California; † University of California – Irvine Medical Center, Department of Emergency Medicine, Orange, California

**Keywords:** cystitis, ketamine, phenazopyridine, methemoglobinemia, case report

## Abstract

**Introduction:**

As ketamine gains traction as an alternative to opiates in the treatment of chronic pain, ketamine-induced ulcerative cystitis is now being recognized as a complication of its use. The first-line treatment is phenazopyridine, an over-the-counter medication for dysuria that historically has been known to cause methemoglobinemia. This report details the case of a patient presenting to the emergency department (ED) with methemoglobinemia.

**Case Report:**

A 66-year-old woman with a complicated medical history presented to the ED with anemia and hypoxia after extended use of phenazopyridine for treatment of ketamine-induced ulcerative cystitis. She was found to have methemoglobinemia secondary to phenazopyridine used to treat her ketamine-induced ulcerative cystitis, a previously undocumented sequelae of chronic ketamine use. She was admitted to the hospital for three days and made a full recovery.

**Conclusion:**

This case highlights the need to suspect ketamine-induced ulcerative cystitis in patients who use ketamine chronically and be judicious in the use of phenazopyridine for symptom management to prevent life-threatening complications.

## INTRODUCTION

The use of opioids as a mainstay for pain control has been falling out of favor in recent years due to the risk of abuse, respiratory depression, and accidental overdose. Ketamine, which acts as an analgesic and has dissociative properties, has gained traction in recent years as comparable to morphine but with more benign side effects.^1^ Historically known as a drug of abuse, ketamine and the risks of chronic use in a controlled setting are not completely understood.^2^ However, there is an emerging awareness of a rare urologic condition termed ketamine-induced ulcerative cystitis.^3^,^4^ The prevalence of this disorder is difficult to appreciate given its recent emergence as a clinical entity.

In patients with ketamine cystitis, phenazopyridine, a common over-the-counter (OTC) medication used for dysuria, is a first-line medication to treat symptoms. However, phenazopyridine is historically known to cause methemoglobinemia. While methemoglobinemia is a rare condition and the incidence due to phenazopyridine use is unknown, the American Association of Poison Control Centers recently reported 1,234 cases of phenazopyridine exposure over the time frame of one year.^5^ Given its accessibility, caution must be used to limit serious reactions. Here, we present a case of a previously unreported combination of complications of chronic ketamine use in a patient presenting with methemoglobinemia secondary to phenazopyridine use for treatment of ketamine cystitis. Despite the novelty of this presentation, the sequelae of difficult-to-diagnose complications of chronic ketamine use warrants discussion.

## CASE REPORT

The patient was a 66-year-old female with past medical history of endometrial carcinoma status post total abdominal hysterectomy (seven years prior), small bowel adenocarcinoma status post resection (12 years prior), and inoperable partial small bowel obstruction. She was being treated for chronic abdominal pain on home ketamine via patient-controlled analgesia pump. Her history was complicated by a recent diagnosis of ketamine-induced cystitis. She presented to the emergency department (ED) from an outside physician for abnormal hemoglobin of 6.1 grams per deciliter (g/dL) (reference range: 12.0–16.0 g/dL).

On evaluation, the patient reported one to two weeks of hematuria without melena, hematochezia, or hematemesis. She reported continued dysuria, frequency, and suprapubic abdominal pain. She also endorsed worsening fatigue and malaise. She denied dyspnea, chest pain, cough, fevers, lightheadedness, or syncopal episodes. Presentation vital signs showed an oxygen saturation of 85% on room air, blood pressure of 113/67 millimeters of mercury (mm Hg), heart rate of 91 beats per minute, and respiratory rate of 18 breaths per minute. She was started on six liters of nasal cannula oxygen without improvement of oxygen saturation.

Of note, the patient had been admitted three weeks prior with dysuria and suprapubic pain. During hospitalization, she was given ceftriaxone for a presumed urinary tract infection without clinical improvement and with negative urine culture. She was ultimately diagnosed with ketamine-induced cystitis, and her pain regimen was modified with the addition of gabapentin, diazepam, venlafaxine, and phenazopyridine. Although asymptomatic, she was incidentally found to be coronavirus disease 2019 (COVID-19)-positive during that admission. At the current visit, the patient reported improvement in dysuria since starting the new pain regimen. She reported that she took her phenazopyridine more frequently than directed, six to eight tablets per day.

On exam the patient was alert and oriented with no signs of trauma, but conjunctival pallor was present. Heart, lungs, and abdominal exam were all normal. Repeat labs in the ED were notable for hemoglobin of 7.4 g/dL, hematocrit of 22.5 (reference range: 36–46%). Venous blood gas revealed a pH of 7.34 (7.35–7.45), partial pressure of carbon dioxide of 50.1 mm Hg (33–45 mm Hg), and partial pressure of oxygen of 38.9 mm Hg (75–105 mm Hg). D-dimer was 690 nanograms per deciliter (ng/dL) (reference range: less than 250 ng/dL), and urinalysis showed moderate hemoglobin, moderate leukocyte esterase, nitrite positive, red blood cell count of 7 per high power field (HPF) (0–3/HPF), and white blood cell count of 32/HPF (0–5/HPF). On imaging a chest radiograph (CXR) showed no abnormalities, and computed tomography angiogram chest was without evidence of pulmonary embolism or other thoracic abnormalities.

The patient’s initial presentation was concerning for COVID-19 given her marked hypoxia and recent positive test on prior hospitalization, although the lack of response to supplemental oxygen and unremarkable CXR made this unlikely. Given the history of increased phenazopyridine use, a methemoglobin level was obtained, showing an elevated level of 11.6% (reference range: 0.0–1.5%). After consultation with the Poison Control Center the patient was started on methylene blue in the ED with near immediate improvement of oxygen saturation to 92–93% on 2–4 liters nasal cannula. She was then admitted to the inpatient telemetry unit and was hospitalized for three days. She was transitioned from oxygen support, and palliative care was consulted for her pain regimen. Phenazopyridine was discontinued, and she was transitioned to her outpatient regimen of gabapentin, diazepam, and venlafaxine.

**CPC-EM Capsule**

What do we already know about this clinical entity?

*Ketamine-induced ulcerative cystitis is a complication of chronic ketamine use, and the first line treatment, phenazopyridine, has been known to cause methemoglobinemia*.

What makes this presentation of disease reportable?

*The intersection of two rare diagnoses, ketamine cystitis and methemoglobinemia, sheds lights on the potential complications of two common analgesic medications*.

What is the major learning point?

*Suspect ketamine-induced ulcerative cystitis in patients with chronic ketamine use and be judicious in the use of phenazopyridine for symptom management*.

How might this improve emergency medicine practice?

*This case highlights that in medically complex patients, common and easily accessible medications such as phenazopyridine may result in life threatening adverse outcomes*.

## DISCUSSION

Ketamine, a derivative of phencyclidine, was first developed in the 1960s and marketed for its anesthetic properties. Recreational use soon gained popularity due in part to its dissociative and psychedelic effects. Although fatal overdoses are incredibly rare, it is not unusual to see sequelae of recreational ketamine in the ED.^6^ The most common presentation in the ED is altered mental status, although abdominal pain, urinary complaints, and dizziness are also seen.^7^ The sustained use of ketamine may also induce elevated liver enzymes and biliary dilation, as well as the newly recognized ketamine-induced ulcerative cystitis. 8

Despite the potential side effects, ketamine continues to gain popularity as a chronic pain treatment. In 2010 the Institute of Medicine released a report estimating that one in three Americans are living with chronic pain and that estimated costs range from $560–635 billion annually.^9^ In response to concerns over the opioid epidemic, alternative treatments are gaining traction. As recently as 2017, several anesthesia and pain management societies adopted consensus guidelines for ketamine use in chronic pain.^10^ They state that despite the lack of large clinical trials, there is sufficient evidence to suggest that ketamine is safe and effective in managing chronic pain. As more patients turn to ketamine as a means of pain control, it is imperative that we understand its pharmacologic properties and potential complications.

Ketamine primarily acts as a noncompetitive N-methyl-D-aspartate (NMDA) antagonist in the central nervous system, most notably in the prefrontal cortex and hippocampus.^10^ Ketamine decreases the frequency of channel opening in these regions, leading to lower levels of neuronal activity. In the absence of ketamine, NMDA receptor activation plays a significant role in cognition, chronic pain, central sensitization, and opioid tolerance.^10^ To a lesser extent, ketamine also acts through the activation of other receptors such as opioid and D_2_ dopamine receptors and through antagonistic effects on muscarinic receptors, sodium, and potassium channels.^10^ While the secondary pathways are poorly understood, they may contribute to analgesia and mood regulation.

The term ketamine cystitis first emerged in 2007 after a series of daily ketamine users presented with dysuria, urgency, frequency, and hematuria with sterile urine cultures.^3^ On computed tomography (CT) imaging these patients displayed evidence of severe inflammation such as bladder wall thickening, decreased capacity, and perivascular stranding, as well as ulcerative cystitis on cystoscopy. Radiologic studies have since correlated these CT findings as well as the occasional presence of hydronephrosis and ureteral wall thickening.^11^ The pathophysiology of urinary tract damage is not well understood and may be secondary to direct or indirect injury. While symptomatic treatment may help to relieve some of the discomfort in the short term, abstinence from ketamine appears to be the best treatment.^3^,^12^

Phenazopyridine is a commonly used OTC medication for the treatment of urinary discomfort. It has historically been associated with methemoglobinemia, although reported cases are rare.^3^ Methemoglobinemia occurs when ferrous iron (Fe^2+^) is oxidized to the ferric state (Fe^3+^), which decreases the oxygen binding capacity.^13^ Clinically this often presents as headaches, fatigue, shortness of breath, and cyanosis, with severe cases having the potential to be fatal. Due to a leftward shift of the oxygen dissociation curve, patients are often hypoxic without improvement on supplemental oxygen, as seen in our patient. Physiologically, there are two mechanisms that allow for reduction of methemoglobin to its ferrous state. The primary mechanism is through a nicotinamide adenine dinucleotide hydrogen-dependent reaction via cytochrome B5 reductase and secondarily through a less active nicotinamide adenine dinucleotide phosphate hydrogen (NADPH)-dependent reaction generated by G6PD.^13^

Methemoglobinemia is either congenital or acquired. Congenital causes are due to cytochrome B5 reductase deficiency and hemoglobin M disease.^13^ Acquired cases are often secondary to a drug reaction. Many medications can induce methemoglobinemia; of note, these include dapsone, benzocaine, nitrates and nitrites, sulfonamides, and, as in our case, phenazopyridine. Diagnosis of methemoglobinemia requires an arterial blood gas with methemoglobin levels. A level of greater than 5% is consistent with the diagnosis of methemoglobinemia, although symptoms are typically observed at higher levels, unless other complications such as anemia or lung disease are present. Treatment with methylene blue is not indicated in levels less than 20% unless individuals are symptomatic.^13^ Levels above 30% are often considered fatal unless treatment is initiated. In our patient, the anemia caused by hematuria from ketamine cystitis provided increased sensitivity to lower methemoglobin levels and clinical hypoxia.

The primary treatment of methemoglobinemia is methylene blue, although for acquired cases at lower levels, discontinuation of the offending agent may suffice. Methylene blue is dosed at 1–2 milligrams per kilogram intravenously over several minutes; it acts as an electron transporter in the NADPH-dependent pathway to reduce methemoglobin.^13^ Often, patient response is rapid, although repeat dosing may be necessary. A major contraindication to methylene blue is G6PD deficiency, which causes severe hemolysis.^14^ In these patients, ascorbic acid (vitamin C) may be used in place of methylene blue, which acts via a similar mechanism. In refractory cases, the use of hyperbaric oxygen and plasma exchange can be considered.^14^

## CONCLUSION

As the use of ketamine increases in both recreational and controlled medical settings, it is important for the emergency physician to consider the diagnosis of ketamine-induced ulcerative cystitis in patients with urinary tract symptoms in the absence of positive urine cultures and to obtain a thorough history of drug use. This case also highlights that in medically complex patients, common and easily accessible medications such as phenazopyridine may precipitate life-threatening complications.
